# Evaluation of the Abbott BinaxNOW rapid antigen test for SARS-CoV-2 infection in children: Implications for screening in a school setting

**DOI:** 10.1371/journal.pone.0249710

**Published:** 2021-04-05

**Authors:** Neeraj Sood, Rashmi Shetgiri, Anna Rodriguez, Dianna Jimenez, Sonia Treminino, Amanda Daflos, Paul Simon

**Affiliations:** 1 Sol Price School of Public Policy, University of Southern California, Los Angeles, California, United States of America; 2 Schaeffer Center for Health Policy and Economics, University of Southern California, Los Angeles, California, United States of America; 3 Los Angeles County Department of Public Health, Los Angeles, California, United States of America; 4 Keck School of Medicine, University of Southern California, Los Angeles, California, United States of America; 5 Dornsife College of Letters, Arts & Sciences, University of Southern California, Los Angeles, California, United States of America; 6 Office of Mayor Eric Garcetti, City of Los Angeles, Los Angeles, California, United States of America; Waseda University: Waseda Daigaku, JAPAN

## Abstract

**Background:**

Rapid antigen tests hold much promise for use in the school environment. However, the performance of these tests in non-clinical settings and among one of the main target populations in schools—asymptomatic children—is unclear. To address this gap, we examined the positive and negative concordance between the BinaxNOW^™^ rapid SARS-CoV-2 antigen assay and an RT-PCR test among children at a community-based Covid-19 testing site.

**Methods:**

We conducted rapid antigen (BinaxNOW^™^) and oral fluid RT-PCR (Curative Labs) tests on children presenting at a walk-up testing site in Los Angeles County from November 25, 2020 to December 9, 2020. Positive concordance was determined as the fraction of RT-PCR positive participants that were also antigen positive. Negative concordance was determined as the fraction of RT-PCR negative participants that were also antigen negative. Multivariate logistic regression models were used to examine the association between positive or negative concordance and participant age, race-ethnicity, sex at birth, symptoms and Ct values.

**Results:**

226 children tested positive on RT-PCR; 127 children or 56.2% (95% CI: 49.5% to 62.8%) of these also tested positive on the rapid antigen test. Positive concordance was higher among symptomatic children (64.4%; 95% CI: 53.4% to 74.4%) compared to asymptomatic children (51.1%; 95% CI: 42.5% to 59.7%). Positive concordance was negatively associated with Ct values and was 93.8% (95% CI: 69.8% to 99.8%) for children with Ct values less than or equal to 25. 548 children tested negative on RT-PCR; 539 or 98.4% (95% CI: 96.9% to 99.2%) of these also tested negative on the rapid antigen test. Negative concordance was higher among asymptomatic children.

**Conclusions:**

Rapid antigen testing can successfully identify most COVID infections in children with viral load levels likely to be infectious. Serial rapid testing may help compensate for limited sensitivity in early infection.

## Introduction

The COVID-19 pandemic has profoundly changed the way we live. It has led to shutdowns of many sectors across the country, including in-person learning in schools. Closure of schools and remote learning has been theorized to lead to worse educational and mental health outcomes compared with in-person instruction and has added immense stress to parents and caregivers [1,2]. One strategy that has been proposed for safer re-opening of schools is rapid COVID-19 testing of students and staff as an adjunct to public health safety measures [3]. Rapid testing allows for quick identification of potentially infectious individuals so that they can be isolated and removed from the school environment. The success of this strategy depends, however, on the accuracy of the rapid test in correctly identifying those who are infectious. Rapid antigen tests hold much promise for use in the school environment. However, they have been approved only for use among symptomatic individuals, primarily adults, in clinical settings. Recent evaluations have examined the effectiveness of these tests for population screening, however the numbers of reverse transcriptase-polymerase chain reaction (RT-PCR) positive children in these evaluations have been limited [4–6]. Thus, the performance of these tests in non-clinical settings and among one of the main target populations in schools—asymptomatic children—is unclear.

To address this gap, we examined the positive and negative concordance between the BinaxNOW^™^ rapid SARS-CoV-2 antigen assay and an oral fluid RT-PCR test among symptomatic and asymptomatic children at a community-based Covid-19 testing site. We analyzed how the positive and negative concordance varied with symptoms and cycle threshold (Ct) values—a proxy for infectiousness [7,8].

## Methods

We conducted this study at a walk-up testing site in Los Angeles County from November 25, 2020 to December 9, 2020. Participants came to the site for Covid-19 testing because they had symptoms or were asymptomatic but seeking testing for possible Covid-19 exposure or other reasons. Families with children less than 18 years of age were recruited for the study. Verbal parental consent and youth assent were obtained on-site and recorded in an electronic database. The ethics committee approved verbal informed consent as the study was deemed low-risk and it was logistically challenging to obtain written informed consent at a busy public testing site. A brief survey was conducted that included demographic information and whether the reason for testing was illness or Covid-19 symptoms, or due to suspected exposure to Covid-19. The RT-PCR test used was a commercial FDA EUA authorized test offered by Curative Labs, Inc. Oral fluid specimens for RT-PCR were self-collected by participants and sample collection was observed by trained Curative staff. Anterior nasal specimens for antigen testing were collected by trained study staff using swab provided in BinaxNOW^™^ and test was performed as per instructions in the kit. Results for the antigen test were read on site by two study staff and classified as positive, negative, or inconclusive. Results were classified as inconclusive if the staff did not agree on the interpretation of test results or both staff agreed that the results were inconclusive. As a quality control measure, each batch of BinaxNOW^™^ tests was tested using the positive control sample provided for each batch. All test kits passed this quality control measure. Participants were instructed to quarantine at home while awaiting RT-PCR results, and those with positive RT-PCR results were instructed to isolate.

The positive concordance of the antigen test was determined as the fraction of RT-PCR positive participants that were also antigen positive. RT-PCR was determined to be positive at a Ct value of less than or equal to 40. The negative concordance of the antigen test was determined as the fraction of RT-PCR negative participants that were also antigen negative. Multivariate logistic regression models were used to examine the association between positive or negative concordance and participant age, race-ethnicity, sex at birth, symptoms and Ct values. It is important to note that the FDA has not authorized test manufacturers to report Ct values to patients or public health agencies. We used Ct values only to assess results of this research study. We also conducted sensitivity analyses to account for potential false negative results on the RT-PCR test. The study was reviewed and approved by the Los Angeles County Department of Public Health Institutional Review Board.

## Results

RT-PCR test results were available for 783 children and 646 adults. Here we focus on the sample of children. Of the 783 children with RT-PCR results, the antigen test was performed on 779 children and 5 children had inconclusive results, yielding a sample of 774 children with valid RT-PCR and antigen results. Table 1 describes the characteristics of children in this sample. The sample was evenly split between males and females as defined by sex at birth. 84.2% of the children in the sample were Hispanic as the testing site was located in a predominantly Hispanic neighborhood. 23.5% of the parents indicated that the reason for testing was because their child was sick or had symptoms that made them think that their child had COVID-19.

**Table 1:** Descriptive statistics for sample of children with valid RT-PCR and antigen test results.

Variable	Sample Size	Percent of Sample
**Sex at birth**		
Male	383	49.5%
Female	385	49.7%
Missing response	6	0.8%
**Race/Ethnicity**		
Non-Hispanic White	30	3.9%
Non-Hispanic Black	11	1.4%
Hispanic	652	84.2%
Other	81	10.5%
**Age Categories**		
5 to 11 years old	503	65.0%
12 to 17 years old	271	35.0%
**Reason for testing**		
Symptomatic	182	23.5%
Asymptomatic	592	76.5%

226 children tested positive on RT-PCR; 127 children or 56.2% (95% CI: 49.5% to 62.8%) of these also tested positive on the rapid antigen test (Table 2). Positive concordance was 64.4% (95% CI: 53.4% to 74.4%) among symptomatic children and 51.1% (95% CI: 42.5% to 59.7%) among asymptomatic children. Positive concordance was higher among children with lower Ct values for the RT-PCR test. For example, positive concordance was 93.8% (95% CI: 69.8% to 99.8%) for children with Ct values less than 25 and 48.8% (95% CI: 41.0% to 56.6%) for children with Ct values greater than 30. Lower Ct values were associated with higher positive concordance even for asymptomatic children. For example, positive concordance was 100% (97.5% CI: 63.1% to 100.0%) for asymptomatic children with Ct value less than or equal to 25 and 71% (95% CI: 48.9% to 87.3%) for asymptomatic children with Ct values between 25.1 to 30.

**Table 2:** Concordance between RT-PCR and antigen test results.

Variable	Sample Size	Percent Concordant	95% Confidence Interval
**RT-PCR Positive**	226	56.2%	49.5% to 62.8%
***Reason for testing***			
Symptomatic	87	64.4%	53.4% to 74.4%
Asymptomatic	139	51.1%	42.5% to 59.7%
***Ct Values***			
Less than or equal to 25	16	93.8%	69.8% to 99.8%
25.1 to 30	37	73.0%	55.9% to 86.2%
Greater than 30	168	48.8%	41.0% to 56.6%
Missing	5	60.0%	14.7% to 94.7%
**RT-PCR Negative**	548	98.4%	96.9% to 99.2%
***Reason for testing***			
Symptomatic	95	95.8%	89.6% to 98.8%
Asymptomatic	453	98.9%	97.4% to 99.6%

548 children tested negative on RT-PCR; 539 or 98.4% (95% CI: 96.9% to 99.2%) of these also tested negative on the rapid antigen test. Negative concordance was higher among asymptomatic children.

Table 3 shows that patient demographics including age, sex at birth and race/ethnicity were not statistically significantly associated with positive concordance.

**Table 3:** Association between patient characteristics and positive or negative concordance between RT-PCR and antigen results.

Variable	Odds Ratio for Positive Concordance	95% confidence interval	Odds Ratio for Negative Concordance	95% confidence interval
**Sex at birth**				
Male	1.22	0.70 to 2.14	0.35	0.08 to 1.55
Female	Reference	-	Reference	-
**Race/Ethnicity**				
Non-Hispanic Black	0.62	0.02 to 17.39	Omitted^#^	-
Hispanic	1.17	0.07 to 19.38	3.71	0.38 to 36.43
Other	1.3	0.07 to 26.00	Omitted^#^	-
Non-Hispanic White	Reference	-	Reference	-
**Age Categories**				
5 to 11 years old	Reference	-	Reference	
12 to 17 years old	1.35	0.75 to 2.43	0.11	0.02 to 0.60
**Reason for testing**				
Symptomatic	1.66*	0.92 to 2.98	0.22	0.06 to 0.88
Asymptomatic	Reference	-	Reference	-
***Ct Values***				
Less than or equal to 25	15.60***	1.99 to 121.91	-	-
25.1 to 30	2.95***	1.32 to 6.58	-	-
Greater than 30	Reference		-	-
Missing	1.44	0.23 to 9.11	-	-

*p-value < 0.10;

**p-value < 0.05;

*** p-value < 0.01.

^#^Participants in these race-ethnicity groups were omitted from the regression because being in these race-ethnicity groups perfectly predicted negative concordance.

Symptomatic children had a higher odds of positive concordance compared to asymptomatic children, however the odds ratio was only significant at the 10% level (Odds Ratio: 1.66, 95% CI: 0.92 to 2.98). Children with Ct values less than 25.0 (Odds Ratio: 15.60, 95% CI: 1.99 to 121.91) and children with Ct values between 25.1 and 30.0 (Odds Ratio: 2.95, 95% CI: 1.32 to 6.58) had much higher odds of positive concordance compared to children with Ct values greater than 30.0. It is important to note that Ct values are measured on a non-linear scale so that one unit change in Ct value is a two-fold change in viral RNA concentration.

The odds of negative concordance were lower among children 12–17 years old compared to children 5–11 years old. The odds of negative concordance were lower among symptomatic children compared to asymptomatic children.

The RT-PCR test used as a comparator for this study might have some false negative results among those who were asymptomatic and where sample collection was not observed by trained personnel [9]. Assuming a false negative rate of about 5% would imply that about 27 of the 548 children who tested negative on the RT-PCR had an active infection and thus were true positives. This would imply 253 true positives in our sample—the 226 who tested positive on RT-PCR plus the 27 false negatives on the RT-PCR. Similarly, under this scenario we would have 521 true negatives—the 548 children who tested negative on RT-PCR less the 27 false negatives. It is possible that some of these false negatives on RT-PCR are among the 9 children who tested positive on the rapid antigen test but tested negative on the RT-PCR. We can create bounds on the true specificity and sensitivity of the rapid antigen test based on assumptions about how many of the false negatives on RT-PCR tested positive on rapid antigen test. If all 9 children who tested positive on rapid antigen in the RT-PCR negative sample were false negatives on RT-PCR then the specificity of the rapid antigen test compared to a gold standard test is 100% and the sensitivity is 53.8% (9+127/253). If on the other hand none of the false negatives on the RT-PCR tested positive on the rapid antigen test, then the specificity compared to gold standard test is 98.3% (539-27/521) and the sensitivity is 50.2% (127/253). Thus, under the assumption that the false negative rate of the RT-PCR test is about 5%, the specificity of the rapid antigen test is between 98.3% to 100% and the sensitivity is between 50.2% to 53.8%. Similarly, under the assumption that we have 54 false negatives on RT-PCR, or a false negative rate of about 10%, our estimates imply a specificity of rapid antigen test compared to gold standard test of 98.2% to 100% and a sensitivity of the rapid antigen test of 45.4% to 48.6%. Although the results are fairly robust to allowing for a false negative rate of up to 10% it is important to note that we do not know the false negative rate of the comparator RT-PCR test used in this study and the false negative rate could be either higher or lower than 10%.

## Discussion

The US Department of Health and Human Services purchased 150 million BinaxNOW^™^ tests to expand testing capacity and started distributing these tests to states on September 28, 2020. A major objective of the deployment of these tests was to safely reopen schools. However, little data was available on the performance of these tests among asymptomatic school age children. In this study we addressed this knowledge gap.

Our results demonstrate moderate positive concordance and high negative concordance of the BinaxNOW^™^ rapid antigen test with RT-PCR among children. Negative concordance is very high among asymptomatic children, suggesting that false positive results among this group are uncommon. However, positive concordance among asymptomatic children was 51%, indicating that one-time testing of asymptomatic children may not be appropriate for diagnostic testing as it will fail to identify roughly half the infections.

However, rapid antigen testing of children for population screening to identify and isolate potentially infectious children shows promise for two reasons. First, positive concordance among asymptomatic children with low Ct values who are likely to be infectious was high. For example, positive concordance for children with Ct value less than or equal to 25 was 93.8%. Second, serial testing can help compensate for the lower sensitivity of this rapid antigen test. Serial testing is critical because one-time antigen tests might not identify asymptomatic children at or shortly after the onset of infection, but serial testing will likely identify these children as they subsequently develop high viral loads and become infectious a few days later [10].

Although deploying serial rapid antigen tests in schools shows promise, some challenges remain. We do not know the optimal frequency of testing. More research is needed to evaluate the extent to which different frequencies of rapid antigen testing in schools enhances safety, reduces outbreaks, and improves continuity of education. The positive predictive value of the rapid antigen test might be low in environments where the prevalence of active infections is low [11], although, the results from a recent study suggest very high specificity in low prevalence asymptomatic populations [12]. Finally, we need to address implementation challenges associated with deployment of serial antigen testing in schools.

The findings of the study should be viewed in light of its limitations. First, we used a commercial RT-PCR test which was authorized by the FDA for testing in symptomatic individuals only. In addition, the FDA issued an advisory about potential false negative results for asymptomatic individuals where sample collection was not observed by trained personnel [9]. Although sample collection in our study was observed by trained personnel it is still possible that the sensitivity of the comparator RT-PCR test used in our study might be lower than other RT-PCR tests. We addressed this limitation by evaluating the robustness of results using alternate assumptions about the false negative rate for the RT-PCR test. In addition, we believe that comparison of BinaxNOW^™^ with the RT-PCR test we used as a comparator is relevant as it is a widely used RT-PCR test in Los Angeles and other jurisdictions. Second, we used anterior nasal swabs for rapid antigen test and oral fluid sample for RT-PCR. Differences in sample collection site might explain some of our findings. Third, we used Ct values as a proxy for viral load; ideally we should have measured infectiousness by ability to culture virus from the specimen, or by contact tracing. Finally, as most schools in Los Angeles were closed during the study period, the study was done in a high prevalence public testing site and not in schools where prevalence is likely lower.

In summary, the study suggests rapid antigen testing can identify most COVID infections in children with viral load levels likely to be infectious and serial rapid testing may help compensate for limited sensitivity in early infection.

## Supporting information

S1 File Dataset for replication studies. (XLSX)
